# Oxidation of Cyclohexanone with Peracids—A Straight Path to the Synthesis of ε-Caprolactone Oligomers

**DOI:** 10.3390/ma15196608

**Published:** 2022-09-23

**Authors:** Jakub Bińczak, Anna Szelwicka, Agnieszka Siewniak, Krzysztof Dziuba, Anna Chrobok

**Affiliations:** 1 Grupa Azoty Zakłady Azotowe, “Puławy” S.A., Al. Tysiąclecia Państwa Polskiego 13, 24-110 Puławy, Poland or; 2 Department of Chemical Organic Technology and Petrochemistry, PhD School, Silesian University of Technology, Akademicka 2A, 44-100 Gliwice, Poland; 3 Department of Chemical Organic Technology and Petrochemistry, Faculty of Chemistry, Silesian University of Technology, Krzywoustego 4, 44-100 Gliwice, Poland

**Keywords:** Baeyer–Villiger oxidation, oligomerization, cyclohexanone, oligo(ε-caprolactone), peracid

## Abstract

During Baeyer–Villiger (BV) oxidation of cyclohexanone with peracids, oligo(ε-caprolactone) (OCL) may be formed. In this work, a two-step one-pot method for the synthesis of OCL involving the BV oxidation of cyclohexanone with peracids and then oligomerization of the resulting ε-caprolactone has been developed. The process was carried out in two solvents: toluene and cyclohexane. Based on the studies, it was determined that the increased temperature (45–55 °C) and the longer reaction time (4 h) favor the formation of OCls. Among the tested peracids (perC_8_-C_12_), perC_10_ turned out to be the most effective oxidant. Moreover, the obtained oligomers were characterized by means of NMR, MS MALDI TOF, and TGA analyses, which made it possible to determine the structure of oligomers (length and terminal groups of the chains). Additionally, the oligomers obtained after the distillation of the reaction mixture were analyzed.

## 1. Introduction

Among lactones, ε-caprolactone (εCL) is an important intermediate in petroleum industrial chemistry. The global production of εCL is approximately 60,000 tons per year. The main application of εCL is the production of poly(ε-caprolactone) (PCL) by a ring-opening polymerization (ROP).

**Scheme 1:**
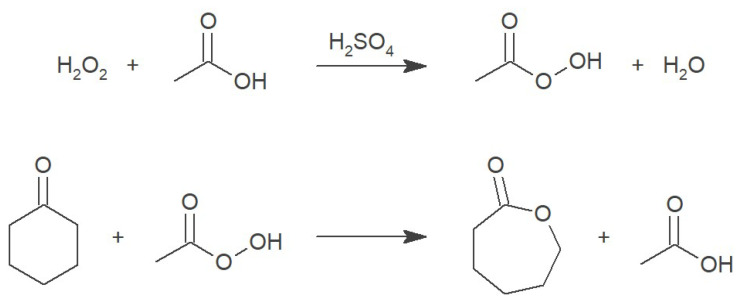
The synthesis of peracetic acid and synthesis of εCL.

One of the methods for the production of εCL uses peracetic acid as an oxidizing agent [1]. Transport and storage of this oxidant is restricted due to its high instability, hence it must be synthesized prior to the cyclohexanone (CNON) oxidation at the same factory. Therefore, the process consists of a two-stage process (Scheme 1). In the first stage peracetic acid is obtained in the oxidation of acetic acid with highly concentrated hydrogen peroxide (>80 wt.%) in the presence of sulphuric acid as a catalyst. Peracetic acid is then purified by vacuum distillation to a 30–55 wt.% concentration. In the second step, a solution of peracetic acid is contacted with cyclohexanone, giving εCL approximately 90% selectivity. The main by-products are 6-hydroxycaproic acid, adipic acid, and oligo(ε-caprolactone) (OCL). Acetic acid, formed during the oxidation from peracetic acid, is recycled to the first oxidation stage.

**Scheme 2:**
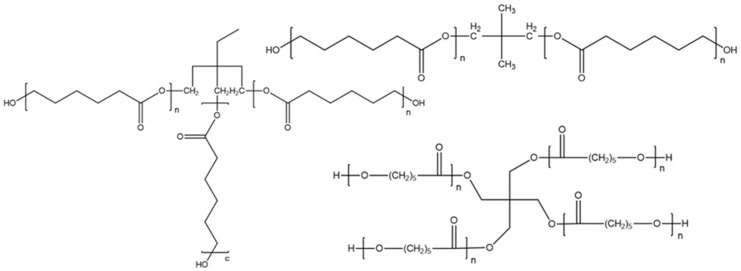
Structures of oligo(ε-caprolactone) with hydroxyl end groups (di-tri- and tetraols).

OCL and PCL are mostly used for biomedical applications and for the production of polyurethane plastics. These materials are characterized by biocompatibility, biodegradability, and bioresorbability, which has been confirmed by numerous studies, including *in vivo* studies on rats [2,3,4,5,6]. Depending on the molecular weight of the polymer, the degradation process can take from several weeks (weights less than 3000 g/mol) to several years. Moreover, PCL shows excellent rheological and viscoelastic properties as well as easy processing. All of this makes it an excellent and perspective material for biomedical applications, in particular controlled drug release systems, production of scaffolds for tissue and bone engineering or in dentistry to fill dental canals [2,3,4,5,6]. PCL and OCL (molecular weights from 400 to 8000 g/mol) are also applied to the production of polyurethanes [2,3,4,5,6], where the presence of hydroxyl groups at the end of the alkyl chain is essential. For this purpose, polyalcohols, such as butanediol, hexanediol, monoethylene glycol, diethylene glycol, glycerin, and pentaerythritol are used as initiators, which allows for obtaining OCL with two, three, or four terminal hydroxyl groups [2,3,4,5,6]. Addition of polyols improves among others processing properties of polyurethanes, mechanical strength (e.g., bending or tensile strength), and resistance to UV radiation. Final materials are used for the production of foams, coatings and thermosetting adhesives [2,3,4,5,6]. The terminal hydroxyl groups in OCL can also be transformed in order to give the oligomer special characteristics, e.g., a product with a so-called shape memory [7]. On the other hand, the use of a special initiator with a multi-arm structure allows for obtaining an OCL with a star-like structure, which can be used as a plasticizer of a modern bioplastic-poly(L-lactide) (Scheme 2) [8,9].

Currently, a limited number of reports regarding the synthesis and properties of oligo(ε-caprolactone) can be found, while poly(ε-caprolactone) is widely discussed [5,10,11,12,13]. The average molecular weight of poly(ε-caprolactone) reached up to 630,000 g/mol [10] with various values of molecular weight distribution (dispersity), including oligomers with a number of repeating units less than 100 [11].

**Scheme 3:**
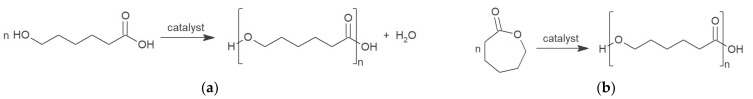
The proposed PCL and OCL synthesis paths: (**a**) polycondensation of 6-hydroxycaproic acid; (**b**) ring-opening polymerization (ROP) of εCL.

Both PCL and OCL can be obtained in polycondensation of 6-hydroxycaproic acid or in ring-opening polymerization of εCL (Scheme 3). Alcohols, such as methanol or glycols, are used as initiators for this process, while catalysts can be divided into three main groups: metal-based, organocatalysts, or enzymes. The properties of obtained polymers depend on a synthesis pathway, used catalysts, and reaction conditions. Exemplary, as a result of polycondensation, a product with a lower molecular weight is usually obtained, while ROP of εCL usually provides better control of the process what results in achieving long-chain polymers with narrower values of dispersity [5,10,11,12,13].

An interesting approach, one-pot synthesis of OCL in cascade enzymatic reactions starting from cyclohexanol was proposed [14]. In this approach, first two enzymes, alcohol dehydrogenase and cyclohexanone monooxygenase, were used for the oxidation of cyclohexanol to cyclohexanone; next, lipase A from *Candida antarctica* was used in chemo-enzymatic Baeyer–Villiger oxidation (BV oxidation) of cyclohexanone to εCL, with further oligomerization of the lactone. The complete conversion of cyclohexanol was observed after 48 h at 30 °C [14].

In this work, a two-step one-pot synthesis of oligo(ε-caprolactone) from cyclohexanone is proposed. In our previous studies, the synthesis of ε-caprolactone using various oxidants (H_2_O_2_, peracids) and catalysts (enzymatic, metal-based and acidic ionic liquids) was developed [15,16,17,18]. Studies on the application of medium-chain peracids have shown that perdecanoic acid was used as an effective oxidant in the Baeyer–Villiger oxidation for the first time (94–99% yield of lactones, 45 min, 35 °C, 5 h) [15]. However, the problem with the selectivity of the product in BV oxidation of cyclohexanone with peracids was observed. It turned out that εCL is prone to oligomerize in the reaction conditions. That brings us to work on formation of OCL via Baeyer–Villiger oxidation/oligomerization in a one-pot manner. Oligo(ε-caprolactone) formed during the two-step process was also characterized, which is extremely important due to the diversity of potential applications of the oligomers, depending on the composition, molecular weight, or the presence of end groups in the oligomer chain.

## 2. Materials and Methods

### 2.1. Materials

Octanoic acid, nonanoic acid, decanoic acid, dodecanoic acid, and 50 wt.% aqueous solution of hydrogen peroxide were purchased from Sigma-Aldrich (Steinheim, Germany) and cyclohexanone was provided by Grupa Azoty Puławy (Puławy, Poland).

### 2.2. GC-FID Analyses

GC-FID analyses were performed on a Perkin Elmer Clarus 500 equipped with a SPB-5^TM^ column (30 m × 0.2 mm × 0.2 µm film). Analysis parameters: injection temperature 250 °C, an FID detector temperature of 250 °C, injection volume 1 µL, carrier gas-helium, a linear flow set as 30 mL/min, air: 450 mL/ min, hydrogen: 45 mL/min, a split ratio of 25:1, an initial temperature of 90 °C with an increasing temperature rate of 5 °C/min → 130 °C with an increasing temperature rate of 5 °C/min → 240 °C (hold for 2 min).

### 2.3. NMR Analyses

Products were analyzed by NMR spectroscopy (Varian NMR system, ^1^H NMR spectra at 600 MHz and 150 MHz for ^13^C NMR). The samples were prepared by dissolving 20 mg of precipitate or 60 µL of the reaction mixture in deuterated chloroform (CDCl_3_).

### 2.4. MS MALDI TOF Analyses

Analyses of oligo(ε-caprolactone) samples were performed on an Axima Performance TOF apparatus, equipped with a positive MALDI ionization source, with a nitrogen laser (337 nm). The samples were dissolved in THF to give 7 mg/mL solutions and then mixed with a 10 mg/mL matrix that was dithranol dissolved in THF. Sodium, lithium, and potassium chloride salts were also added at a concentration of 0.05 M each.

### 2.5. Thermogravimetric Analyses (TGA)

Oligomer samples (approximately 10 mg) were subjected to thermogravimetric analysis on a Mettler Toledo TGA TGA851e thermobalance apparatus. The samples were heated in the range of 25–800 °C with a temperature increase of 10 °C/min with Al_2_O_3_ as a standard in a dynamic nitrogen atmosphere at a flow of 60 mL/min.

### 2.6. Synthesis of Peracids

All peracids were synthesized according to the procedure described in [19]: 8.6 g of decanoic acid dissolved in 21.5 g of 95% sulfuric acid was placed in a tall beaker with a capacity of 250 mL, and the obtained yellow solution, while being intensively stirred with a mechanical stirrer, was cooled in an ice bath to a temperature of 10 °C. After the temperature had stabilized, 5.1 g of a 50% aqueous solution of hydrogen peroxide was added dropwise, taking care that the temperature of the mixture did not exceed 30 °C. After the oxidant was added dropwise, the mixture was stirred at 1000 rpm for 50 min. At the end of this time, ice with water (approximately 150 mL) was slowly added to the beaker and the peracid crystallized as a white solid. The contents of the beaker were transferred to a separatory funnel, washing the beaker with 100 mL of cold water, followed by extraction with diethyl ether (5 × 30 mL). The organic phase was then washed with 20 mL of water and dried over MgSO_4_. The precipitate was filtered off and the filtrate was evaporated without heating the bath to give crude peracid, which was then recrystallized with petroleum ether (10 mL/1 g of crude product) at −18 °C. The product was then filtered off under vacuum, washed with a little chilled petroleum ether to give the product in 90% yield with 100% purity.

The other peracids were synthesized analogously, and the following parameters were used for the syntheses: 21.6 g of octanoic acid (C_8_), 21.5 g of 95% sulfuric acid, 15.1 g of 50% aqueous hydrogen peroxide solution, crystallization temperature −18 °C, 85% yield, 100% purity; 18.0 g nonanoic acid (C_9_), 20.0 g 95% sulfuric acid, 12.1 g 50% aqueous hydrogen peroxide solution, crystallization temperature −35 °C, yield 90%, purity 100%; 10.0 g of dodecanoic acid (C_12_), 21.5 g of 95% sulfuric acid, 5.1 g of 50% aqueous hydrogen peroxide solution, crystallization temperature −18 °C, 85% yield, 100% purity.

### 2.7. Synthesis of Oligo(ε-Caprolactone)

Cyclohexanone, and, for safety reasons, part of cyclohexane or toluene (20 wt.%), which were the reaction solvents, were introduced into the oxidation reactor and preheated to 35 °C. Then peracid was added to the reactor with the rest of the organic solvent in a molar ratio to the ketone of 0.5–2 eq. The mixture was heated to a given temperature (45–55 °C), at which the reaction mixture was then maintained for 4 h. The reaction mixture was directed to the distillation column, where cyclohexane, cyclohexanone, εCL, decanoic acid were successively distilled. Then, an appropriate amount of methanol (approximately 30 mL per 4 g cyclohexanone) was added to the distillation residue (mainly the oligomer) and left at −18 °C to precipitate oligo(ε-caprolactone), which was then filtered under vacuum and washed with 5 mL methanol per 1 g of cyclohexanone. If necessary, a further recrystallization from methanol was applied.

## 3. Results

### 3.1. OCL Synthesis and Analysis

**Scheme 4:**
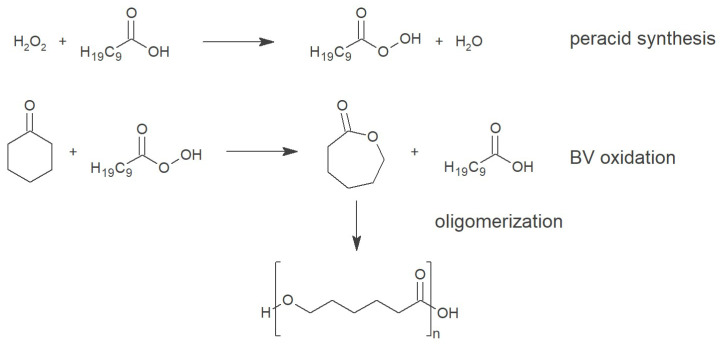
The synthesis of perdecanoic acid and one-pot BV oxidation/oligomerization synthesis of OCLs.

In the preliminary studies, the tests of BV oxidation of cyclohexanone by various peracids were performed (Scheme 4). Peracids were synthesized according to the literature [15]. Linear carboxylic acids (C_8_, C_9_, C_10_, C_12_) were stirred with 95% of sulfuric acid at 10 °C, and next 50% H_2_O_2_ was added dropwise, keeping the temperature below 30 °C. After the reaction, water with ice was added in order to cool the mixture and precipitate the solid peracid. Then, diethyl ether was added to dissolve the peracid, and the formed biphasic system was separated. The organic phase was next collected and dried under anhydrous MgSO_4_. The product was recrystallized from petroleum ether at −18 °C and then the corresponding peracid was obtained via vacuum filtration with >99% purity (iodometric titration).

**Figure 1:**
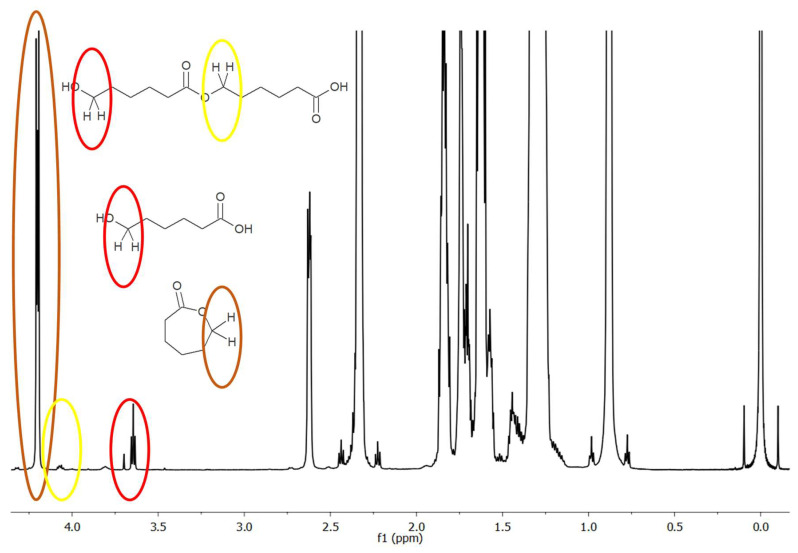
^1^H NMR signals of εCL and oligomers (spectrum of exemplary reaction mixture).

The main goal of the preliminary studies was to identify the reaction conditions that promote the formation of OCL. To compare the effect of the solvent on the CNON conversion and the kind of obtained products, toluene, cyclohexane or excess of cyclohexanone were used. Various peracids were introduced to the process to determine their influence on the amount and kind of synthesized OCL. The experiments were carried out at 25–55 °C up to 4 h with molar ratio of cyclohexanone to peracid (CNON/peracid) ranging from 1:0.5 to 1:2. The conversion of cyclohexanone was analysed by GC, while the selectivity to OCL was calculated by ^1^H NMR spectroscopy (Figure 1).

As shown in Table 1 the reaction time and temperature have a significant influence on the selectivity of the process. After 4 h of reaction at 45 or 55 °C in cyclohexane or toluene, using perC_10_ as an oxidant, almost complete conversion and selectivity to OCLs were achieved. It is assumed that under these conditions, in the presence of peracid most likely the ring opening of ε-caprolactone, formation of the hydroxy acid and then its oligomerization successively occurred. This confirms that the oligomers can be obtained by a one-pot two-step BV oxidation-oligomerization process (Scheme 4). Extending the reaction time from 60 min to 240 min resulted in an increase in selectivity to OCLs at 45 or 55 °C in cyclohexane or toluene, using perC_10_. In turn, only 9% selectivity towards OCLs was reached at 25 °C in cyclohexane after 1 h, while at 45 °C it was already 62%. However, the reaction cannot be carried out at temperatures higher than 65 °C due to the thermal decomposition of the peracid. The most effective peracid for proposed one-pot OCL synthesis is perC_10_ (58% selectivity to OCL after 60 min), which is additionally in agreement with the safety of the process. In our previous work the stability tests for several peracids (perC_6_-C_12_) were performed concerning sensitivity to mechanical impulse (shock and friction), electrical (spark), and thermal sensitivity (temperature and heat of decomposition) and perC_10_ was selected as the safest peracid [15]. For other peracids, selectivity to OCLs did not exceed 19% after 60 min. Regardless of the solvent, the use of an excess of peracid in relation to CNON increased both the conversion of CNON and the selectivity to OCLs. For toluene, 58% selectivity to OCLs was achieved with a two-fold molar excess of peracid (90% CNON conversion), while an equimolar amount of CNON:perC_10_ allowed for obtaining only 28% selectivity and 74% CNON conversion after 60 min. A similar phenomenon was observed for cyclohexanone. The kind of solvent did not strongly affect the conversion nor selectivity. Preliminary studies also confirmed that the use of CNON in excess, and thus the elimination of additional solvents, leads to only traces of OCLs (up to 5%).

**Figure 2:**
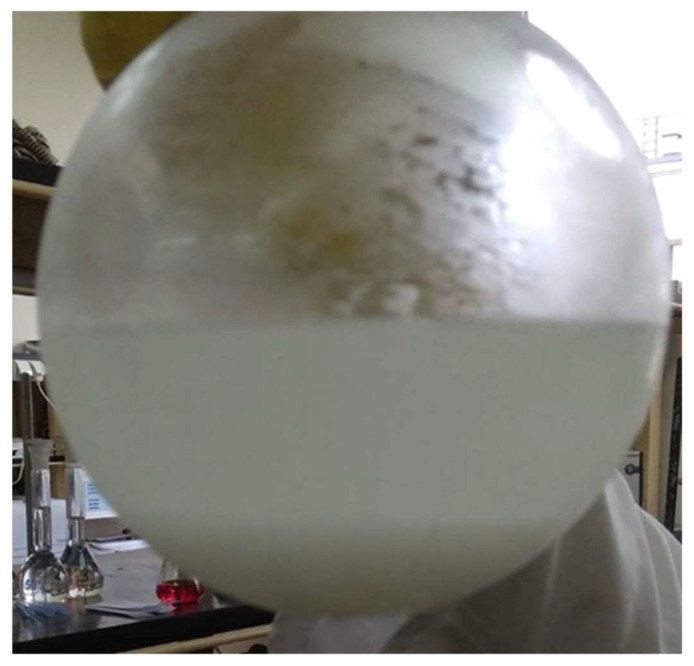
The OCLs after crystallization.

In order to determine the structure of obtained OCLs, the oxidation of cyclohexanone was scaled up four times. The experiments were carried out with deficiency of peracid (molar ratio CNON:perC_10_ 1:0.5) at 55 °C in cyclohexane or in toluene, approaching the almost 100% selectivity to OCLs. The reaction mixtures were first concentrated using a rotary evaporator to get rid of the solvent and unreacted cyclohexanone. Next, methanol was added, the flask was cooled and kept overnight at −18 °C to precipitate the OCLs. The procedure was repeated twice, achieving the purity of the product over 99% (Figure 2). For the characterization of the product, ^1^H NMR spectroscopy and MS MALDI TOF spectrometry were exploited.

**Figure 3:**
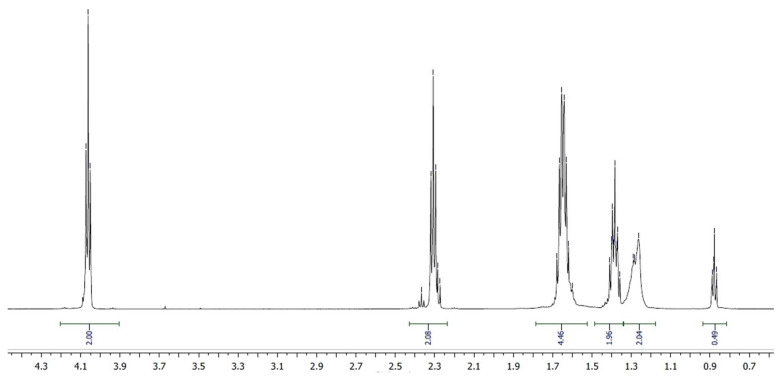
^1^H NMR of OCL isolated from reaction carried out in toluene.

It is worth noticing that the esterification of OCLs with decanoic acid during the reaction as well as with methanol during crystallization of OCLs can occur. In the ^1^H NMR spectrum (Figure 3) for the process carried out in toluene, the triplet visible at approximately 4.05 ppm corresponds to the CH_2_ group protons that are attached to the ester group by a covalent bond with oxygen. The higher the ratio of this signal to the signals at approximately 3.65 ppm, the longer the OCLs, assuming no other end groups in the molecule. In this case, a very weak signal present in the range of 3.50–3.70 ppm indicates that there are a few compounds with a CH_2_ group attached to a free -OH group. The alcohol chain ends, however, may have been esterified with decanoic acid, which would confirm the presence of an asymmetric signal at approximately 4.05 ppm and the methyl group signal of the aliphatic acid chain at 0.85 ppm. It is also possible that cyclic oligomers are present in the sample, which would also not give signals in the range of 3.50–3.70 ppm with significant signal amplification at 4.05 ppm. Therefore, the NMR spectrum is not suitable for unambiguous information about the chain length of the oligomer and the number of oligomers of different chain lengths in the sample.

**Figure 4:**
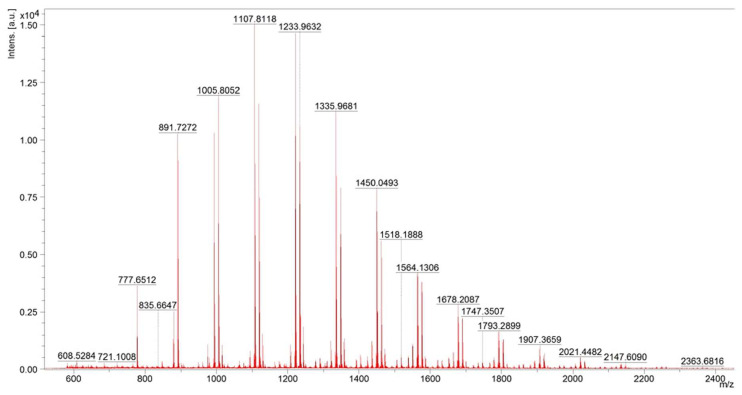
MS MALDI TOF of OCL isolated from reaction carried out in toluene.

Hence, MS MALDI TOF analysis of OCLs was performed. In the spectrum (Figure 4), the most intense signal is 1108 m/z (one repeating unit of oligo(ε-caprolactone) possesses a molecular mass of 114 Da). This signal is a part of one set of peaks and does not correspond to a cyclic oligomer (multiplying the repeating unit n times by 114 Da), which suggests the presence of linear oligomers with end groups. It was assumed that OCLs were singly ionized and m/z ratio corresponds to the mass in Da. On the other hand, the signal corresponding to the mass 1006 m/z does not belong to the same sets of peaks, and therefore it corresponds to the oligomers obtained as a result of incorporation of successive repeating units into the compound with a different end group. In-depth analysis of the spectrum confirms that there were two major sets of oligomers with different chain lengths in the spectrum. The signal from the longest oligomer 2364 Da corresponds to 19 repeating units in the oligomer molecule terminated with an -OH group esterified with decanoic acid, after ionization of carboxyl group by sodium coming from the glass under analysis conditions. Analysis of signals in the spectrum, together with the ^1^H NMR spectrum, showed that the second set of oligomers most likely corresponds to the same end groups but is ionized with potassium coming from the matrix. The result of the MS MALDI TOF analysis explains why no signals from CH_2_ groups at the free -OH group were observed in the ^1^H NMR spectrum—such groups do not exist in the sample due to the esterification with decanoic acid.

**Scheme 5:**
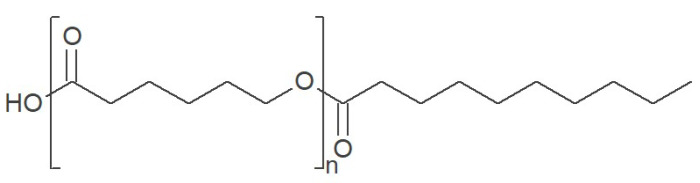
Oligomers isolated after the reaction in various solvents, n < 24 for cyclohexanone; n < 20 for toluene.

MS MALDI TOF analysis of the OCLs isolated from the reaction carried out in cyclohexane (Figure S1) was performed. In the obtained spectrum, the most intense signal equals 1222 m/z, which corresponds to 9 repeating units terminated with a sodium-ionized carboxyl group and hydroxyl group esterified with decanoic acid. The final signal corresponds to a mass of 2821 Da as assigned to the 23 repeating units of oligo(ε-caprolactone). The ^1^H NMR spectrum (Figure S2) confirms the results of the MS MALDI TOF analysis. Based on the results of these analyses, the proposed structure of OCLs is presented in Scheme 5.

**Figure 5:**
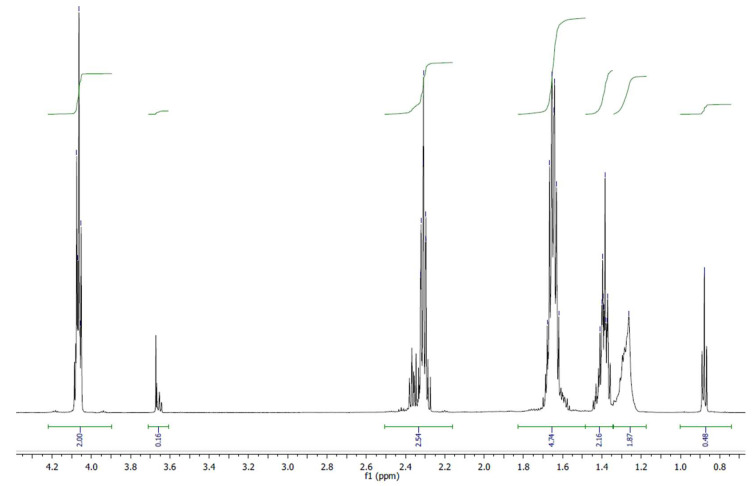
^1^H NMR analysis of OCL for CNON:perC_10_ molar ratio 1:0.5.

Next, the influence of the molar ratio of CNON:perC_10_ ranging from 1:0.5 to 1:2 on the structure of OCLs was studied. Due to the negligible effect of the solvent the processes were carried out in cyclohexane as it is characterized by a higher vapor pressure and thus easier working up of the reaction mixture. ^1^H NMR spectra of products from these processes indicate that oligo(ε-caprolactone) with a similar structure was obtained (Figure 5, Figures S3 and S4). The ratio of the signals at approximately 4.05 ppm and 3.65 ppm reached similar values (approximately 13.5). The average molecular weight of the obtained oligomers was approximately 1500 Da, however it is not known whether the signals corresponded to one or several sets of OCLs nor whether there were any other compounds in the sample that could give similar signals (such as cyclic oligomers or the presence of the group esterified with decanoic acid in the sample). The signal at 4.05 ppm is not a symmetrical triplet, confirming the presence of a decanoic acid ester as a chain end in at least one of the resulting oligomer series. The presence of decanoic acid or its ester is clearly evidenced by the signal at approximately 0.85 ppm, coming from the methyl group. On the basis of the obtained spectra, it is suspected that the peracid to substrate ratio at the stage of the oxidation process does not significantly affect the structure of the obtained OCLs, which must be confirmed by further MS MALDI TOF analyses.

**Figure 6:**
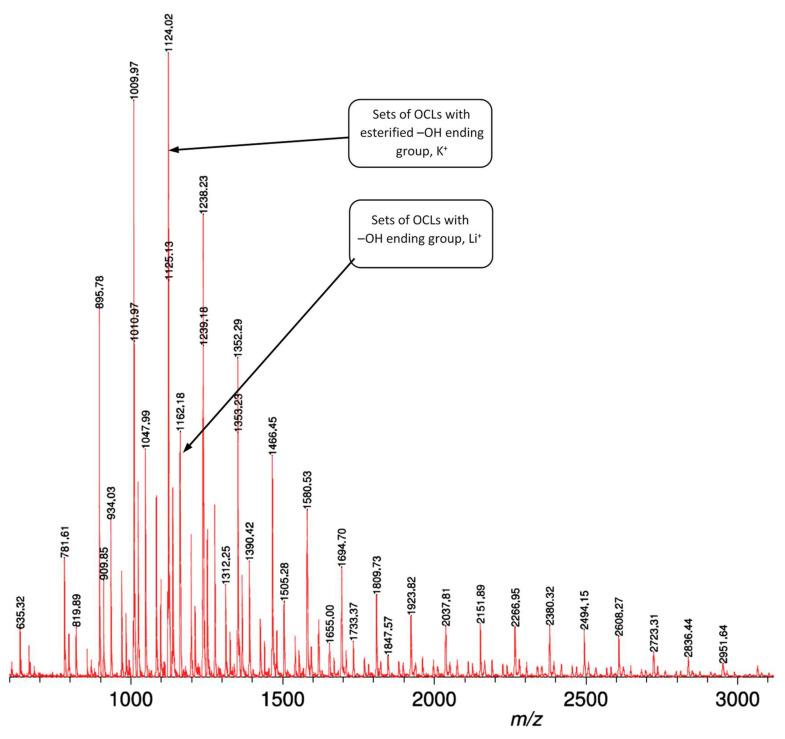
MS MALDI TOF analysis of OCL for CNON:perC_10_ molar ratio 1:0.5.

MS MALDI TOF of OCLs were performed in the range 600–4000 m/z (Figure 6, Figures S5 and S6)). The average molecular weight is in each case approximately 1100–1300 Da, which is a slightly lower value than that estimated by interpreting the ^1^H NMR spectra. This suggests that in MS spectra it is necessary to find signals from OCLs with a free terminal -OH group, and that cyclic oligomers could also arise, which would increase the integral value of the proton signal in the ^1^H NMR spectrum at approximately 4.05 ppm in relation to the proton signals at approximately 3.65 ppm. The higher the ratio of CNON:perC_10_ used in the oxidation step, the shorter the oligomers obtained. This is most likely due to a lower concentration of peracid in the reaction mixture leading to incorporation of successive units into the structure of already formed oligomers than into the structure of monomer and short-chain oligomers that start the next chain. Apparently, there is not enough decanoic acid to esterify the -OH group and to complete the formed oligomer chain. In the case of a higher concentration of perC_10_ in the mixture, subsequent peracid molecules take part in the oxidation of cyclohexanone faster, which increases the reaction course and hence short-molecule oligomers are formed. Oligomers can be terminated more rapidly with ester groups due to a higher amount of decanoic acid in the reaction system, which is the process that completes the incorporation of subsequent mers into the oligomer structure. Moreover, two main sets of oligomers were observed in the sample coming from the process conducted with a deficiency of perC_10_. Less peracid in the system leads to OCL being obtained more selectively. The difference in the structure of OCL obtained with the perC_10_ deficiency process compared to the others was observed. For these OCLs, one sets of peaks corresponds to a group of oligomers terminating with an esterified -OH group from one side and with a carboxyl group ionized with potassium from another one. Another set corresponds to oligomers with a free -OH group at the end of the chain and a lithium-ionized carboxyl group. The most intense signal in this spectrum—1124 Da—corresponds to the oligomer concerning 8 repeating units and belongs to the sets of peaks ionized by potassium. In the case of samples from processes carried out with a higher molar ratio of perC_10_:CNON, an additional set of peaks coming from cyclic oligomers was observed. The other two series correspond to an OCLs terminated with an -OH group esterified with decanoic acid or non-esterified and with a carboxyl group ionized with lithium or potassium. The only difference is that shorter oligomers were obtained in a process using an excess of peracid. The similar ratio of the signals at 4.10 to 3.65 were caused by the fact that in the first case the longest oligomers were obtained, and in the second case cyclic oligomers did not give a signal at 3.65 ppm in the ^1^H NMR spectrum, which increased the value of the estimated molecular weight of the oligomers. In the case of the oxidant deficiency process, the highest molecular weight of the oligomer (2952 Da) observed on the spectrum corresponds to the 24 repeating units incorporated into OCL, with the -OH ending group esterified with decanoic acid. In the case of the oxidation process carried out with an equimolar amount of perC_10_, the highest molecular weight of the oligomer is 2509 Da, which corresponds to the 22 repeating units incorporated into cyclic OCL, and in the case of the oxidation process carried out with a double excess of oxidant, the OCL with the highest mass was obtained (1694 Da), which corresponds to only 13 repeating units in the structure of OCL with an -OH esterified ending group in the molecule. Hence, it is possible to obtain εCL oligomers more selectively, which form linear esterified or non-esterified molecules or cyclic oligomers, depending on the implemented conditions.

**Scheme 6:**
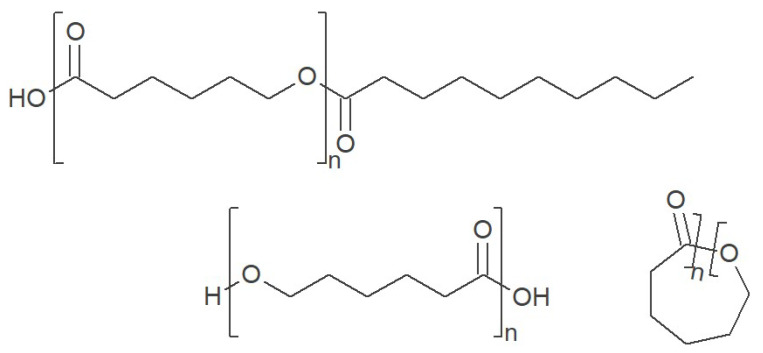
Oligomers isolated after the oxidation of CNON.

**Scheme 7:**
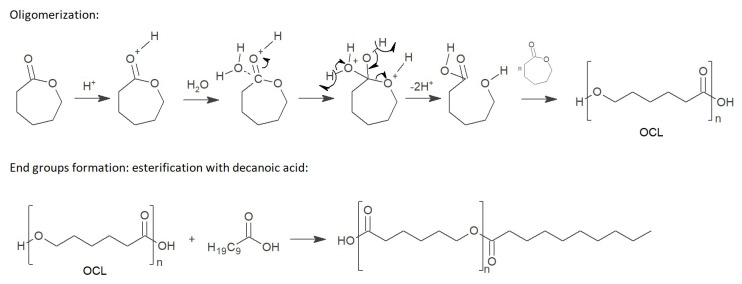
Proposed oligomerization mechanism.

Therefore, the following conclusions followed from performed experiments: (a) CNON: perC_10_ ratio affects both: the type and molecular weight of the obtained εCL oligomers; (b) the higher concentration of perC_10_ in the reaction system, the shorter oligomers were obtained after the same time; and (c) in the case of conducting the cyclohexanone oxidation process with an insufficient amount of oxidant, cyclic oligomers were hardly obtained. The structure of the created OCLs were shown in Scheme 6 and a proposed oligomerization mechanism is presented in Scheme 7.

### 3.2. Analysis of OCLs Separated during Distillation

In the industrial oxidation of cyclohexanone by perC_10_ acid to εCL, the product is isolated by vacuum distillation. Therefore, in the next step, it was checked whether exposing the post-reaction mixture to a high temperature during distillation under reduced pressure influences the type of end groups, dispersibility, and average molecular weight of the byproduct OCLs. The oxidation process was carried out in toluene (molar ratio CNON:perC_10_ 1:0.6, 55 °C, 4 h). After the reaction, the mixture was sent to a distillation process to evaporate the solvent at a pressure of 500 mbar. The mixtures were then preheated to 100 °C, the pressure was lowered to approximately 15 mbar, and next mixtures were heated gradually to 200 °C, collecting the rest of the solvent, cyclohexanone, and a small amount of the lactone and 6-hydroxycaproic acid at 10 mbar. To characterize the OCLs after distillation, the distillation residue was dissolved in methanol and left overnight. The obtained oligomers were then analyzed by NMR, MS MALDI TOF, and TGA.

**Figure 7:**
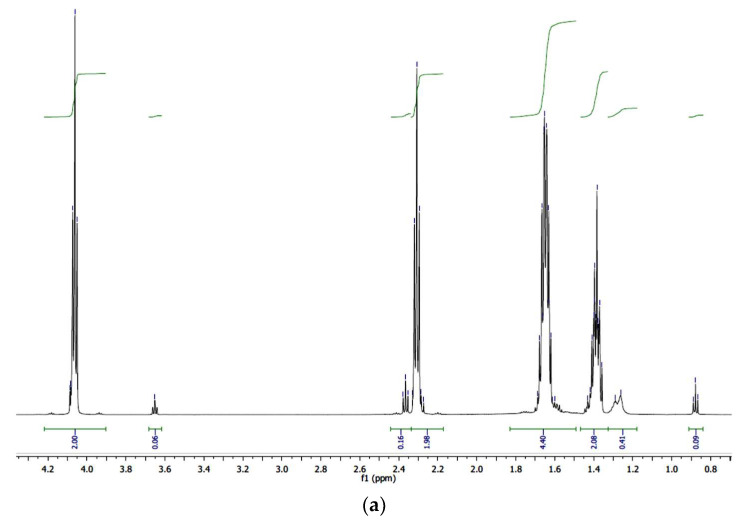

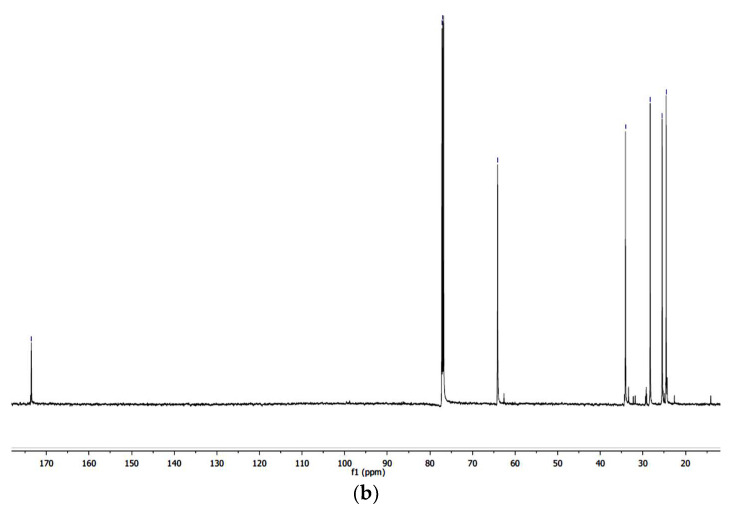
(**a**) ^1^H NMR; (**b**) ^13^C NMR, of the OCls obtained after distillation of the reaction mixture from the oxidation of CNON in toluene.

Analyzing the ^1^H NMR spectrum (Figure 7a) of the precipitate obtained after distillation of mixture from the process carried out in toluene, it can be seen that its main component is oligo(ε-caprolactone). The characteristic peaks at approximately 4.10 ppm and 3.65 ppm (signal ratio approximately 34) may indicate that only a small amount of chain terminated with free -OH groups are present in the oligomer. Most likely decanoic acid is incorporated into the chain endings. Such a high value of this ratio may be also due to the presence in the system a significant amount of oligo(ε-caprolactone) in the cyclic form or oligomers with longer chains than those obtained so far because these compounds stayed at high temperature for a much longer time. Based on the results of the experiments described above, it is predicted that at this molar ratio of CNON: perC_10_ acid, such a high value of signal ratio is mainly due to the presence of the high-molecular weight oligomers and not cyclic compounds. Moreover, it is apparent that decanoic acid is most likely incorporated into the chain ends, which would not be possible with only cyclic oligomers. ^13^C NMR spectrum (Figure 7b) also shows that the basic component of the obtained precipitate is oligo(ε-caprolactone) but gives no additional information.

**Figure 8:**
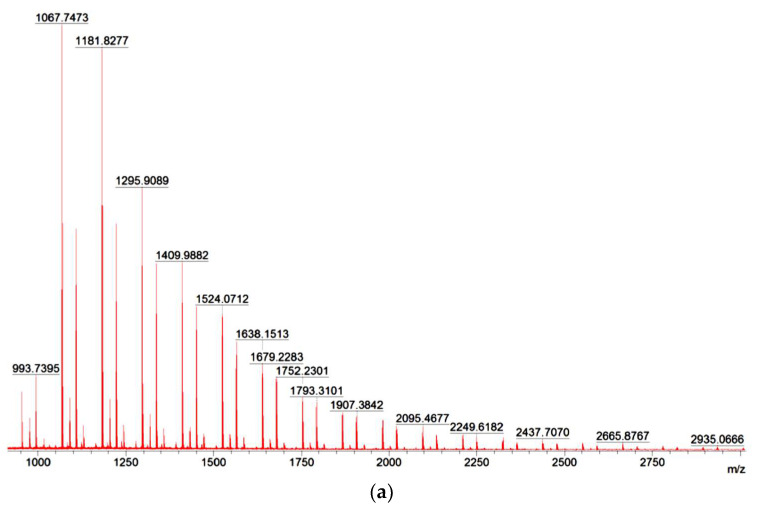

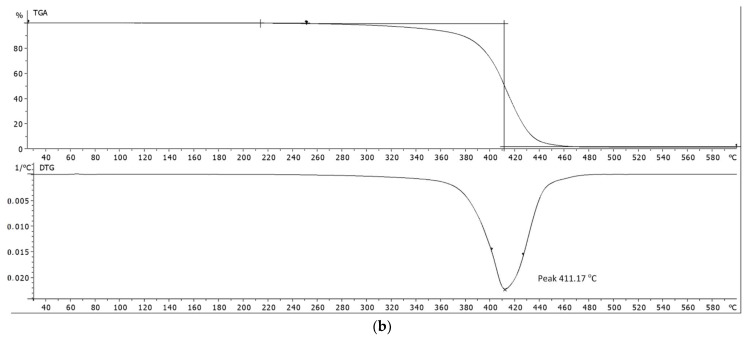
(**a**) MS MALDI TOF; (**b**) TGA-DSC spectra of the OCls obtained after distillation of the reaction mixture from the oxidation of CNON in toluene.

MS MALDI TOF analysis (Figure 8a) shows that the sample was composed of oligomers with different end groups in the chain, giving two sets of signals. The most intense 1068 Da signal suggests that it is a 9 repeating units incorporated into OCLs terminated with -OH and -COOH groups ionized with sodium. The signal of 2935 Da belongs to the same series of signals, corresponding to 24 repeating units terminated in the same way. The second signal set visible on the spectrum, but present in a much smaller amount, is the sodium-ionized oligo(ε-caprolactone) peaks with the -OH group esterified with decanoic acid, which includes, for example, the signal at 1107 m/z corresponding to the 8 repeating units in the structure of oligomer. The spectrum shows that oligomers with a molecular weight of up to 3000 Da were obtained in the process. The interpretation of the MS MALDI TOF spectrum is therefore consistent with the preliminary analysis of the NMR spectra.

TGA-DSC analysis (Figure 8b) was also performed. The obtained spectra show that the loss of mass in the sample only occurs in the temperature range from approximately 310 to 480 °C and it corresponds to the range of oligo(ε-caprolactone) presented in the literature [20,21]. The absence of other effects and the shape of the curve indicate that decanoic acid is present in the sample as a chain ending rather than free acid. Therefore, it can be concluded that the obtained oligo(ε-caprolactone) consists of two series of oligomers with chains terminated with carboxyl groups and an alcohol or ester, with a high purity and an average molecular weight in the range of 1200–1500 Da.

**Scheme 8:**
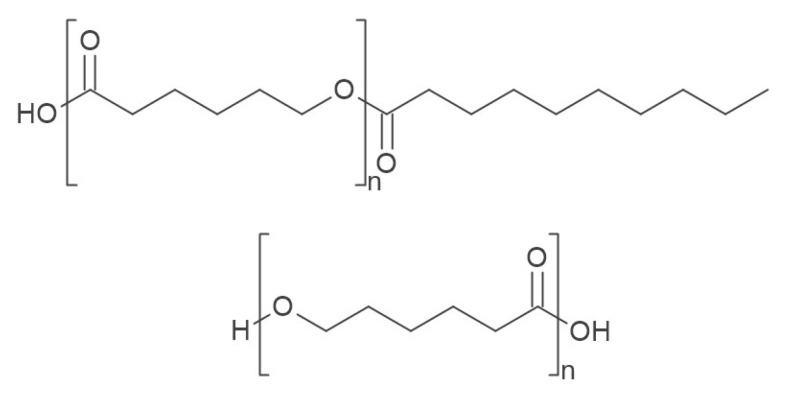
Oligomers isolated after distillation of mixture from the oxidation of CNON.

The experiments of post-reaction mixtures after distillation under conditions similar to the industrial show that: (a) it is possible to obtain an oligo(ε-caprolactone) mixture with a molecular weight of up to 3000 Da, (b) at a high temperature, mainly linear oligomers terminated with a free -OH group or an -OH group esterified with decanoic acid, and (c) it is possible to isolate oligo(ε-caprolactone) with a purity of approximately 99% with a satisfactory efficiency (69% of the mass of cyclohexanone introduced into the process). Scheme 8 shows the structures of oligo(ε-caprolactone) isolated from the performed process.

## 4. Conclusions

An effective method for εCL oligomers synthesis was developed. The method is based on the Baeyer–Villiger oxidation of cyclohexanone with peracids and the oligomerization of the resulting ε-caprolactone. The highest yield of OCLs was obtained using a temperature in the range of 45–55 °C, a reaction time of 4 h, and perC_10_ acid as an oxidant. It has been shown that the course of the process is not significantly influenced by the solvent used: toluene or cyclohexane. Then, an effective method of isolating εCL oligomers from the post-reaction mixture was developed by precipitation from solution by adding methanol and purification of oligo(ε-caprolactone) by crystallization. The developed technique of separating oligomers is easy to perform, undoubtedly has application potential, and does not consume a significant amount of additional media.

In order to unequivocally determine the structure, purity, and thermal stability of the obtained εCL oligomers, it is enough to use three analytical techniques: NMR, MS MALDI TOF, and TGA. The information obtained as a result of MS MALDI TOF and ^1^H NMR analyses allow for obtaining information on the structure of the synthesized oligomers, while TGA analysis allows for assessing the purity of a given sample, as well as the thermal stability of the obtained oligomers, which in total gives the full characteristics of OCLs. This information can be used to determine the direction of application of oligomers, e.g., in biomedicine, or as plasticizers, or in combination with cross-linking agents, such as polyalcohols, for the production of compounds with specialized applications.

## 5. Patents

Chrobok, A.; Szelwicka, A; Sitko, M.; Tadasiewicz, D.; Schimmelpfennig, L.; Dziuba, K.; Morawiec-Witczak, M. Method of preparing oligo(ε-caprolactone). Patent PL 239463, 10 September 2021.

## Figures and Tables

**Table 1:** One-pot BV oxidation/oligomerization in various solvents with selected peracids.

CNON/Peracid Molar Ratio	Solvent	Temperature, (°C)	Time, (min)	α of Ketone, (%)	S to OCLs, (%)
1:2 (perC_8_)	toluene	45	60	92	10
1:2 (perC_9_)	toluene	45	60	96	19
1:2 (perC_10_)	toluene	45	30 60	79 90	19 58
1:2 (perC_12_)	toluene	45	60	89	7
1:1 (perC_10_)	toluene	45	60 240	74 95	28 95
1:0.5 (perC_10_)	toluene	55	240	50	95
1:2 (perC_10_)	cyclohexane	25	60	52	9
1:2 (perC_10_)	cyclohexane	45	60	91	62
1:1 (perC_10_)	cyclohexane	55	240	99	98
1:0.5 (perC_10_)	cyclohexane	55	240	50	98
1:1 (perC_10_)	cyclohexanone	45	60	92	5

Reaction conditions: cyclohexanone (0.1 g, 1 mmol), peracid, solvent (1 mL), 1200 rpm; α—conversion determined by GC; S—selectivity to OCLs determined by ^1^H NMR. All experiments were repeated three times, and the results in each case differed by less than 1%.

## Data Availability

Not applicable.

## Supplementary Materials

The following supporting information can be downloaded at: https://www.mdpi.com/article/10.3390/ma15196608/s1, Figure S1: MS MALDI TOF of OCL isolated from reaction carried out in cyclohexane; Figure S2:1H NMR of OCL isolated from reaction carried out in cyclohexane; Figure S3: 1H NMR analysis of OCL obtained in the reaction with CNON:perC10 molar ratio 1:1; Figure S4: 1H NMR analysis of OCL obtained in the reaction with CNON:perC10 molar ratio 1:2; Figure S5: MS MALDI TOF analysis of OCL obtained in the reaction with CNON:perC10 molar ratio 1:1; Figure S6: MS MALDI TOF analysis of OCL obtained in the reaction with CNON:perC10 molar ratio 1:2.
