# Supplementary material for: Oxidation of Cyclohexanone with Peracids—A Straight Path to the Synthesis of ε-Caprolactone Oligomers
Source: Materials (Basel). 2022 Sep 23;15(19):6608. doi: 10.3390/ma15196608 (PMC9571306; doi:10.3390/ma15196608)
Supplement: Supplementary file 1 [file materials-15-06608-s001.zip › materials-1908067-supplementary.pdf]

Supplementary Materials

Oxidation of cyclohexanone with peracids – a straight path to the synthesis of ε-caprolactone oligomers

Jakub Bińczak [1,2], Anna Szelwicka [3], Agnieszka Siewniak [3], Krzysztof Dziuba [1] and Anna Chrobok [3,*]

1 Grupa Azoty Zakłady Azotowe „Puławy" S.A., Al. Tysiąclecia Państwa Polskiego 13, 24-110 Puławy, Poland
2 Department of Chemical Organic Technology and Petrochemistry, PhD School, Silesian University of Technology, Akademicka 2A, 44-100 Gliwice, Poland
3 Department of Chemical Organic Technology and Petrochemistry, Faculty of Chemistry, Silesian University of Technology, Krzywoustego 4, 44-100 Gliwice, Poland
* Correspondence: anna.chrobok@polsl.pl; Tel.: +48 32 2371032

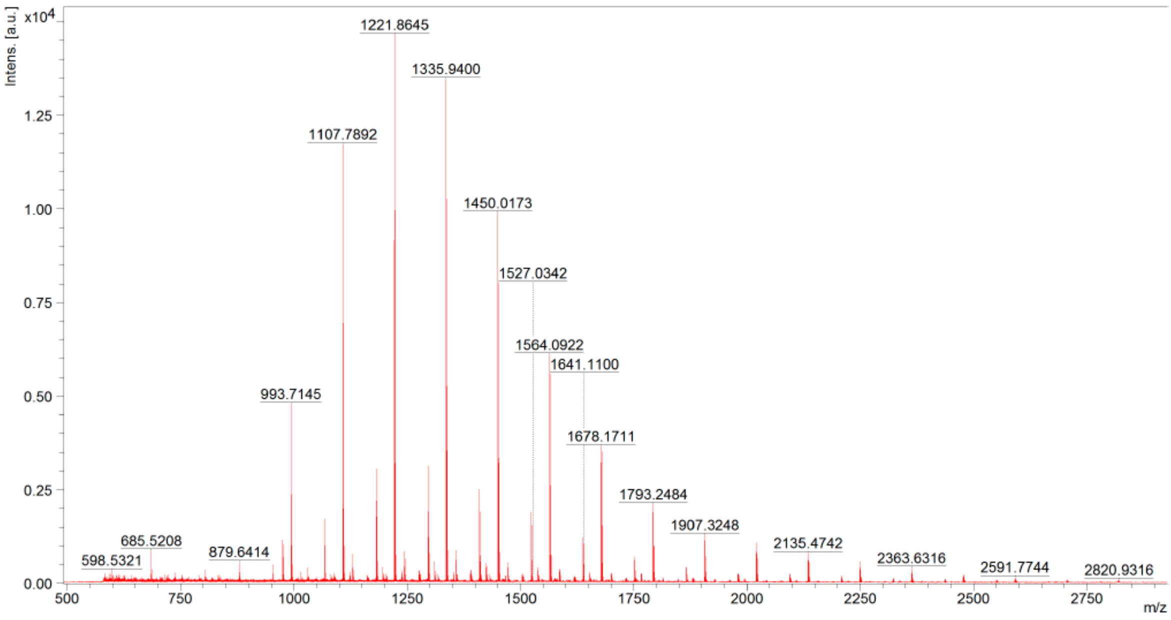

Figure S1. MS MALDI TOF of OCL isolated from reaction carried out in cyclohexane.

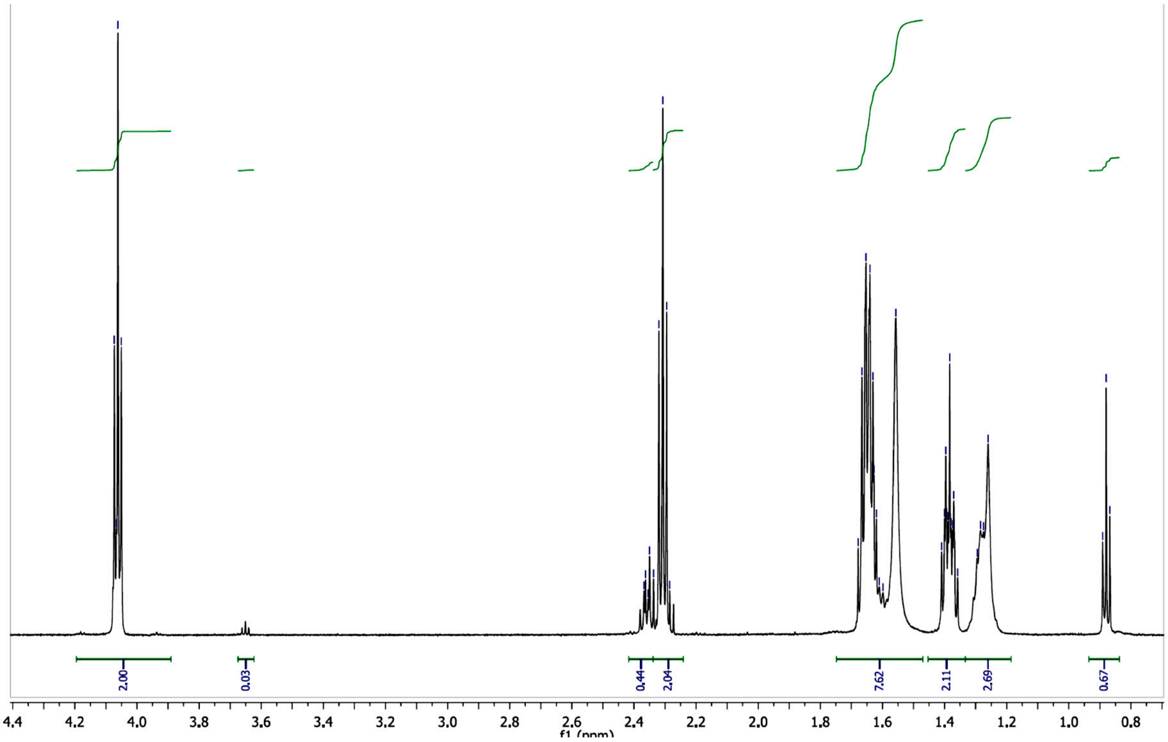

Figure S2. ^{1}H NMR of OCL isolated from reaction carried out in cyclohexane.

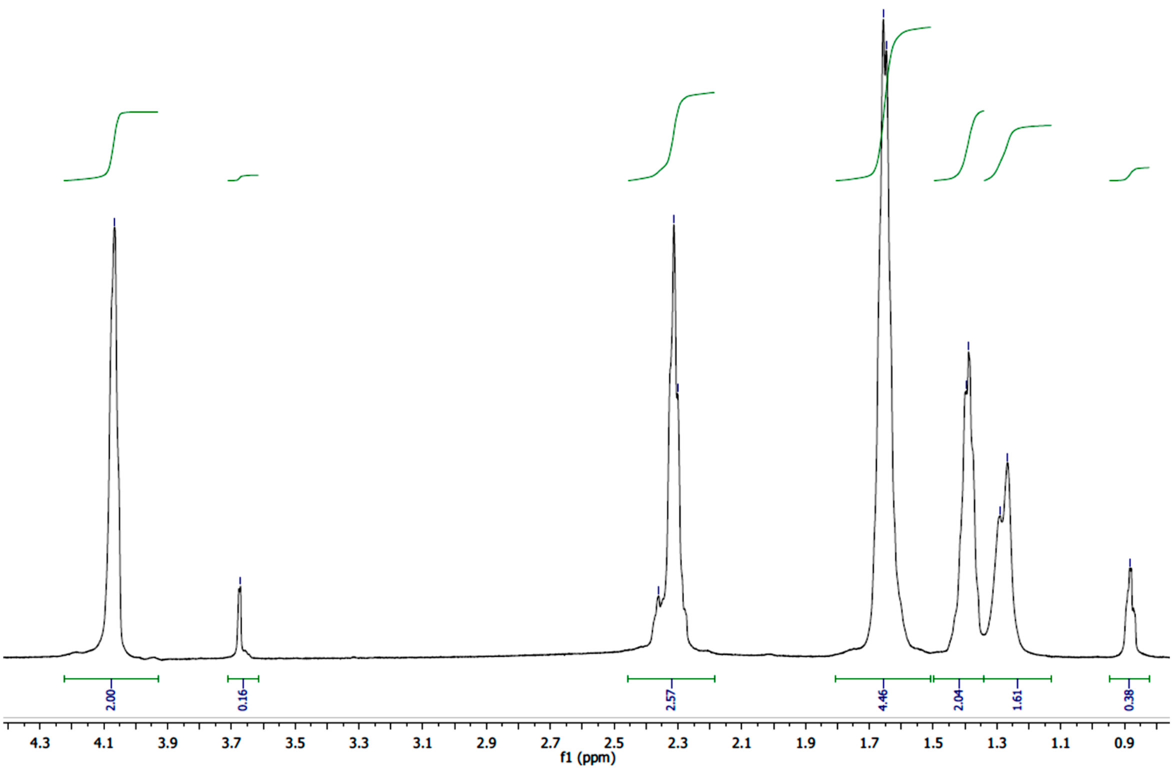

Figure S3. ^{1}H NMR analysis of OCL obtained in the reaction with CNON:perC$_{10}$ molar ratio 1:1.

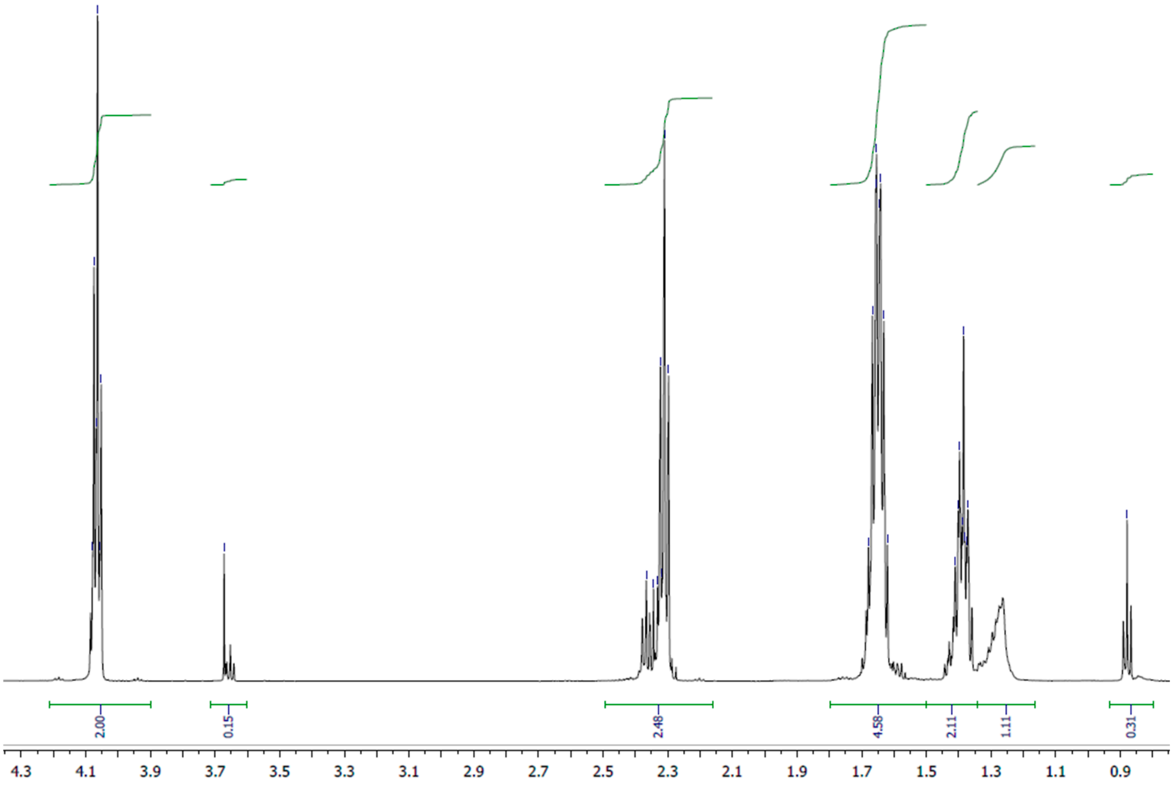

Figure S4. ^{1}H NMR analysis of OCL obtained in the reaction with CNON:perC$_{10}$ molar ratio 1:2.

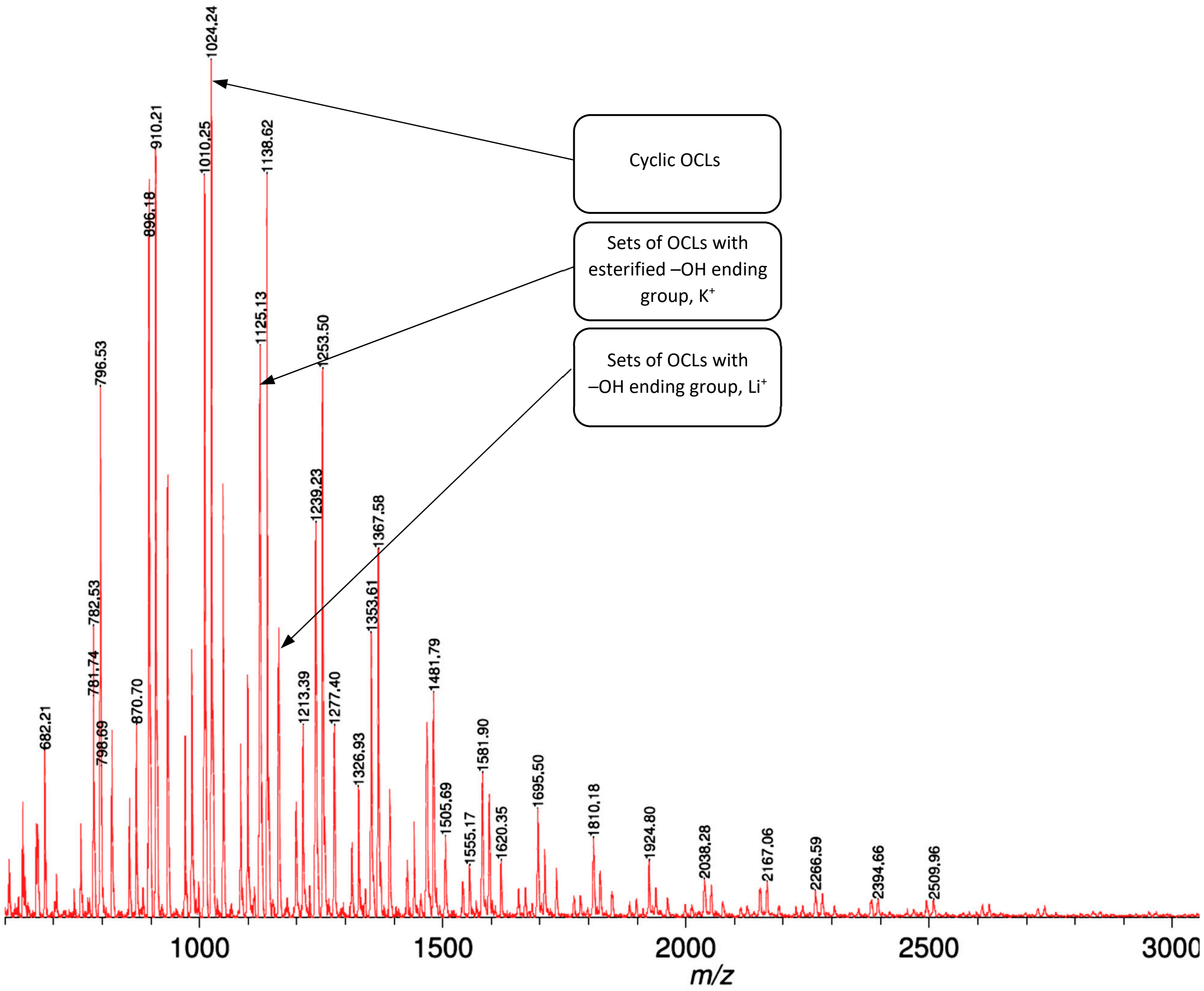

Figure S5. MS MALDI TOF analysis of OCL obtained in the reaction with CNON:perC$_{10}$ molar ratio 1:1.

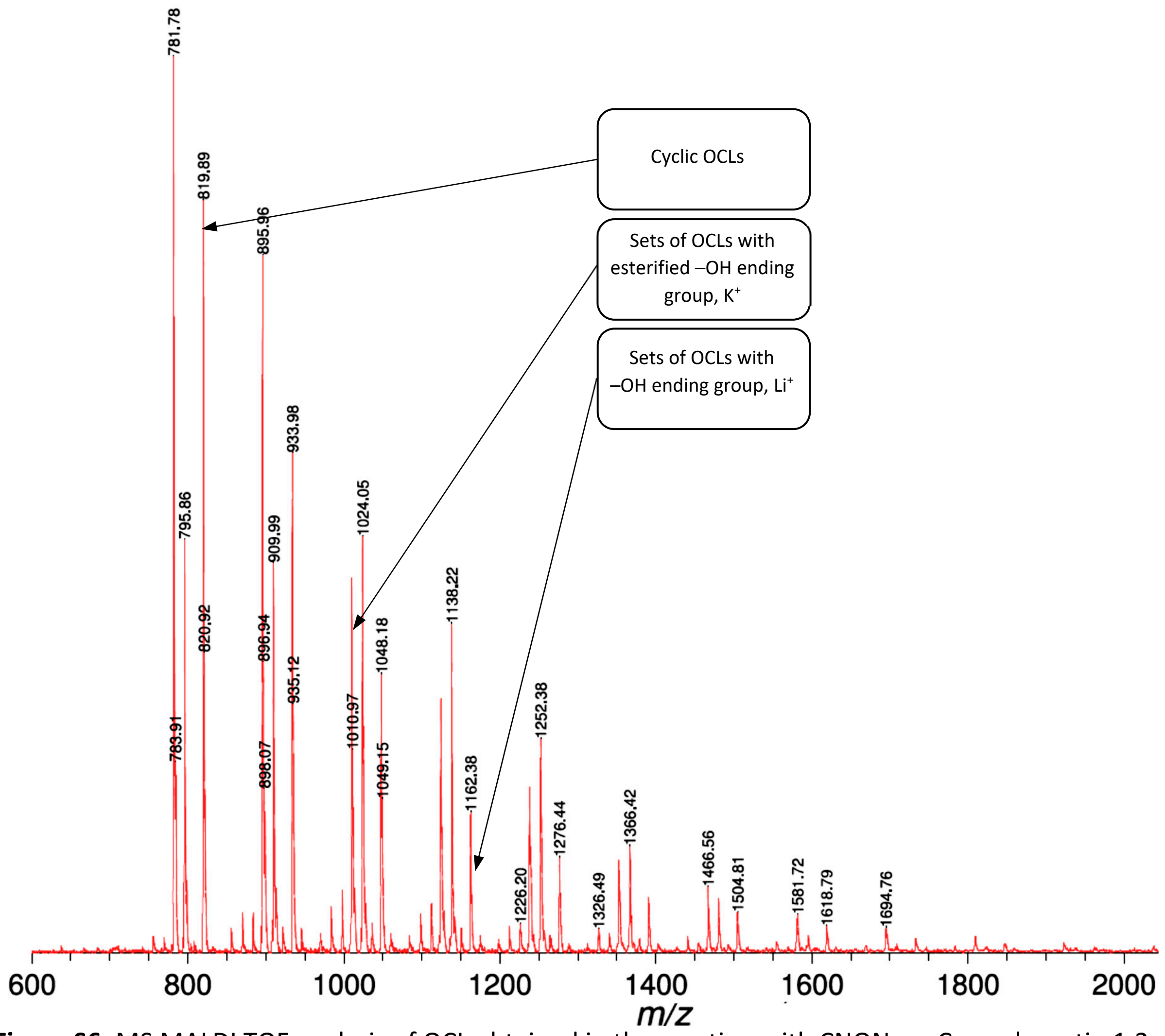

Figure S6. MS MALDI TOF analysis of OCL obtained in the reaction with CNON:perC_{10} molar ratio 1:2.
